# Cryopreservation in Trehalose Preserves Functional Capacity of Murine Spermatogonial Stem Cells

**DOI:** 10.1371/journal.pone.0054889

**Published:** 2013-01-22

**Authors:** Yong-An Lee, Yong-Hee Kim, Bang-Jin Kim, Byung-Gak Kim, Ki-Jung Kim, Joong-Hyuck Auh, Jonathan A. Schmidt, Buom-Yong Ryu

**Affiliations:** 1 Department of Animal Science and Technology, Chung-Ang University, Ansung, Gyeonggi-Do, Korea; 2 Department of Food Science and Technology, Chung-Ang University, Ansung, Gyeonggi-Do, Korea; 3 Department of Science, Spokane Community College, Spokane, Washington, United States of America; University of Connecticut Health Center, United States of America

## Abstract

Development of techniques to isolate, culture, and transplant human spermatogonial stem cells (SSCs) has the future potential to treat male infertility. To maximize the efficiency of these techniques, methods for SSC cryopreservation need to be developed to bank SSCs for extended periods of time. Although, it has been demonstrated that SSCs can reinitiate spermatogenesis after freezing, optimal cryopreservation protocols that maximize SSC proliferative capacity post-thaw have not been identified. The objective of this study was to develop an efficient cryopreservation technique for preservation of SSCs. To identify efficient cryopreservation methods for long-term preservation of SSCs, isolated testis cells enriched for SSCs were placed in medium containing dimethyl sulfoxide (DMSO) or DMSO and trehalose (50 mM, 100 mM, or 200 mM), and frozen in liquid nitrogen for 1 week, 1 month, or 3 months. Freezing in 50 mM trehalose resulted in significantly higher cell viability compared to DMSO at all thawing times and a higher proliferation rate compared to DMSO for the 1 week freezing period. Freezing in 200 mM trehalose did not result in increased cell viability; however, proliferation activity was significantly higher and percentage of apoptotic cells was significantly lower compared to DMSO after freezing for 1 and 3 months. To confirm the functionality of SSCs frozen in 200 mM trehalose, SSC transplantation was performed. Donor SSCs formed spermatogenic colonies and sperm capable of generating normal progeny. Collectively, these results indicate that freezing in DMSO with 200 mM trehalose serves as an efficient method for the cryopreservation of SSCs.

## Introduction

Postnatal mammalian males have the capacity for germ cell division and sperm production throughout adult life through an organized, complex process called spermatogenesis [1]–[2]. The cellular foundation of this process is the spermatogonial stem cells (SSCs) that have the ability to self-renew or differentiate into cells committed to become spermatozoa [3]–[4]. Coupled with techniques such as SSC culture and transplantation, isolation and preservation of SSCs can serve as an efficient mechanism to perpetuate an individual male's germ line [4]–[5] for reproductive management of livestock and endangered species, creation of transgenic organisms, and the treatment of human male factor infertility.

Techniques have been developed for the isolation, enrichment, transplantation, and characterization of SSCs from mammals including rodents [6]–[9] and livestock [10]–[11]. Once isolated, SSCs can be maintained for extended periods of time by long-term cell culture or cryopreservation. Culture methods have been developed for mammals including rodents and humans; however, these methods are time intensive, costly, and have the potential to alter the SSC population [12]–[17].

Cryopreservation of SSCs has the potential to serve as a more efficient method than culture for the long-term preservation of SSCs. After freezing, thawed SSCs retain the ability to establish spermatogenesis and differentiate into spermatozoa capable of generating offspring without any apparent genetic errors after transplantation into recipient testes [4], [18]–[19]. These results indicate that at least some SSCs survive after freezing. The initial methods of cryopreservation were surprisingly simple compared to the difficulties associated with the freezing of spermatozoa [20]–[22] and were readily applied to SSCs of other species, including rats, hamsters, cattle, non-human primates, and humans [23]–[32]. Experiments have also shown that testis tissue survives cryopreservation [33]. However, despite the potential for cryopreservation demonstrated in these initial studies, the techniques used were the same that were used for somatic cells and established cell lines, and few attempts to optimize the cryopreservation technique for SSCs were conducted [23]–[32]. Additionally, the cell populations in these original studies were not enriched for SSCs and the effect of cryopreservation on SSC viability or proliferative capacity was not evaluated.

The objective of this study was to determine the efficiency of cryopreservation of SSCs suspended in trehalose. Inclusion of trehalose in cryopreservation media enhances cell viability [34]–[35] increases colony formation of human hematopoietic stem cells [36] and improves the viability of differentiated mammalian germ cells after cryopreservation [37]–[38]. To evaluate the different treatments, freezing medium containing dimethylsulfoxide (DMSO) or DMSO with varying concentrations of trehalose, freezing protocols were examined for cell recovery, viability, in vitro proliferation capacity, and ability to re-establish spermatogenesis and fertility in recipient animals.

## Materials and Methods

### Ethics statement

All animal procedures were approved by the Animal Care and Use Committee of Chung- Ang University (Permit Number: 11-0038) in accordance with the *Guide for the Care and Use of Laboratory Animals of the National Institutes of Health*. All efforts were made to minimize animal suffering.

### Isolation and culture of testis cells enriched for SSCs

Unless otherwise stated, all reagents were purchased from Sigma-Aldrich (St. Louis, MO, USA). Experimental animal lines were originally purchased from The Jackson Laboratory (Bar Harbor, Maine, USA). Donor testis cells (containing the SSC population) for analysis of viability, proliferation capacity, apoptosis, and SSC quantification were obtained from 6–8 day-old F1 progeny from C57GFP bred into the DBA/2 background (C57GFP X DBA). Because of the background of recipient males for fertility analysis, donor cells for fertility experiments were obtained from 6–8 day-old C57BL/6-TgEGFP (C57GFP) mice. These mouse strains express the enhanced green fluorescent protein (EGFP) in all cells allowing for identification of donor cells in non-EGFP recipients. Isolation of cell populations enriched for SSCs was performed as previously described [14] with minor modification. Briefly, fresh testes were placed in Dulbecco's Phosphate Buffered Saline (DPBS; Invitrogen, Grand Island, NY, USA) and decapsulated. Seminiferous tubules were then incubated in a 2∶1 solution of 0.25% trypsin-EDTA in 1 mM EDTA (Invitrogen) and 7 mg/ml DNAse I (Roche, Basel, Switzerland) in DPBS at 37°C for 5 min, followed by enzyme inactivation by the addition of fetal bovine serum (FBS; Hyclone, Thermo Scientific, Logan, UT, USA) equivalent to 10% of the initial reaction volume. Following digestion, testis cell suspensions were filtered through a nylon mesh with 40 μm pores (BD Biosciences, San Jose, CA, USA), and cell viability was determined by trypan blue exclusion. Viability after digestion and filtration was always greater than 95%. After filtration, cells were centrifuged at 600 g for 7 min at 4°C and the pellet was resuspended in Dulbecco's Modified Eagle Medium (DMEM; Invitrogen) (containing 10% FBS, 2 mM L-glutamine, 0.1 mM β-mercaptoethanol, 100 U/ml penicillin, and 100 μg/ml streptomycin) to 5×10^6^ cells/ml. To remove erythrocytes and cellular debris from the testis cells, 10×10^6^ cells were overlaid on 2 ml of 30% Percoll solution and centrifuged. The resulting pellet was resuspended and magnetic-activated cell sorting (MACS) using anti-Thy-1 antibody microbeads (Miltenyi Biotech, Auburn, CA, USA) was performed to isolate Thy-1 positive cells which are enriched for spermatogonial stem cells. Following isolation of Thy-1^+^ cells, germ cell cultures were generated by placing 0.1×10^6^ Thy-1^+^ cells per well of 12-well culture plates containing mitotically inactivated STO (SIM mouse embryo-derived thioguanine- and ouabain-resistant) feeder cells. Cultures were maintained in mouse serum free medium (mSFM) containing 10 ng/ml GDNF (R&D Systems, Minneapolis MN, USA), 75 ng/ml GFRα1 (R&D Systems), and 1 ng/ml basic fibroblast growth factor (FGF2; BD Biosciences) as previously reported [14]. Cell cultures were passaged 1∶2 or 1∶3 every 7 days and medium was replaced every 2–3 days.

### Cryopreservation

Four weeks after germ cell culture initiation, cultured germ cells, which were enriched for SSCs compared to initial testis digests, were isolated by digestion in 0.25% trypsin and suspended at 5×10^5^ cells/mL in freezing media consisting of MEM alpha (Invitrogen) with 10% dimethyl sulfoxide (DMSO) and 10% FBS without additional supplements (basal freezing medium) or basal freezing medium with the addition of 50 mM, 100 mM or 200 mM trehalose in 1.8 ml cryovials (Corning, Midland, MI, USA). Cryovials were placed in a Nalgene freezing container (Nalgene, Rochester, NY, USA) containing 100% isopropyl alcohol that provided a 1°C/min cooling rate when stored at −80°C. After overnight freezing, cryovials were transferred to liquid nitrogen for long-term storage.

### Viability and proliferation analyses

After freezing for 1 week, 1 month, or 3 months, frozen cells (regardless of freezing protocol) were thawed by incubation in a 37°C water bath for 2.5 minutes and evaluated for viability and proliferation potential. After thawing, cells were diluted 1∶10 with MEM alpha containing 10% FBS in a dropwise manner. Cell viability was determined by trypan blue exclusion. Viability analysis was conducted after thawing because all cells were not recovered after washing (Figure S1).

To evaluate proliferative ability of the SSC enriched germ cells, thawed cells were cultured in mSFM as described above for 1 week. A 1 week culture period was chosen to allow for complete loss of dead cells, acclimation to culture of live cells, and because doubling rate of similarly aged non-frozen cells in our system is approximately 1 week. In order to compare proliferation capacity across cryopreservation treatments as well as to determine the overall effects of cryopreservation on the proliferation capacity of thawed germ cells enriched for SSCs, proliferation capacity was normalized to non-cryopreserved cells. Thawed and non-cryopreserved cells were cultured at a density of 5×10^5^ cells per well of a 12 well plate. Non-cryopreserved germ cell cultures, used for normalization, were from established proliferating germ cell cultures. After 1 week in culture, cells were removed from culture wells by trypsin digestion and GFP positive cells were counted. The cell proliferation among treatments was evaluated by counting cells after 1 week in culture and normalizing to the proliferation of non-cryopreserved cells using the following equation:
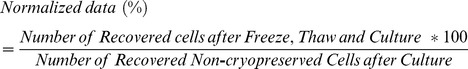



### Germ cell transplantation

Germ cell transplantation was conducted to evaluate the effects of cryopreservation directly on the SSC population. The only way to definitively quantify the number of SSCs in a given cell population is the germ cell transplantation assay. BALB/c/Bk1 immunodeficient nude mice (Nara Biotech., Seoul, Republic of Korea) that were treated with 40–44 mg/kg busulfan at 6 weeks of age to deplete endogenous spermatogenesis, were used as recipients for C57GFP X DBA donor cells to directly quantify SSCs in specific treatment groups. Because immunodeficient nude mice do not efficiently generate offspring after germ cell transplantation, 12–14 or 24 day-old W mice, that lack endogenous spermatogenesis due to a mutation in the c-kit receptor tyrosine kinase gene [39], were used as recipients for C57GFP donor cells to evaluate the effects of freezing treatments on the ability of frozen SSCs to generate complete functional spermatogenesis. After recipient preparation, thawed cells that had been frozen for 3–6 months and cultured for 1 week were concentrated and stained with trypan blue to visualize injection. Recipient mice were anesthetized i.p. with 75 mg/kg ketamine and 0.5 mg/kg medetomidine and injected with door cells into the testes through efferent ducts as previously described [40]. Approximately 8 µl (5×10^6^ cells/ml) and 2–4 µl (50×10^6^ cells/ml) of donor cell suspension was introduced into the testes of nude and W mice respectively resulting in the filling of 80% of surface seminiferous tubules.

### Analysis of stem cell activity and fertility

Two months after transplantation of C57GFP X DBA germ cells into recipient nude mice, recipients were euthanized and testes were recovered and decapsulated. To quantify SSCs, the numbers of fluorescent donor colonies, confluent groups of donor cells 1 mm or greater in length were counted for each recipient testis using fluorescence microscopy as previously described [41]. Colony numbers were expressed as the number of colonies per 10^5^ cells transplanted using the following equation:
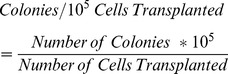



Colony numbers were also expressed as the number of colonies per number of cells recovered after culture because of potential effects of cryopreservation on SSC proliferation, using the following equation:
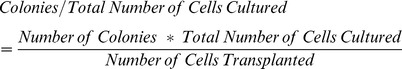



To confirm the extent of donor spermatogenesis in recipient nude testes, testes were obtained 4 months after transplantation, cryosectioned and visualized histologically using fluorescence microscopy.

One to two months after transplantation of C57GFP germ cells into recipient W mice, recipients were housed with two wild type B6 adult females and allowed to mate. Pups born 90 days (to ensure that progeny were from transplanted SSCS) after housing with females were analyzed visually for the presence of GFP to determine if frozen SSCs had the capacity to differentiate into fertile sperm.

### Analysis of germ cell apoptosis

To determine the effect of freezing cells in trehalose on apoptosis, percentage of apoptotic cells was determined immediately after thawing and after a 12-hour post-thaw culture period. Cells were processed using an Annexin V-Phycoerythrin (PE) apoptosis kit (BD Biosciences) with a modified protocol. Briefly, thawed cells were washed with DPBS twice and resuspended at 2×10^5^ cells/200 µl in 1x binding buffer. Ten µl of Annexin V-PE was added and the cells were incubated for 15 min at room temperature in the dark. Propidium iodide (PI) buffer was added to a final concentration of 5 µg/mL and cells were placed on ice. Apoptotic cell populations were determined with BD FACS Diva software analysis of data obtained using a FACSAria flow cytometer (BD Biosciences, Center for Research Facilities, Chung-Ang University) equipped with Cell Quest software.

### Statistical analysis

Statistical analysis was conducted using SPSS version 18 software (SPSS Inc., Mechanicsburg, PA, USA). Analysis of variance (ANOVA) and Tukey's honestly significant difference (HSD) test was used to test for differences between treatment groups for viability, proliferation, apoptosis, and colony number. Differences were considered significant if p<0.05. Unless otherwise stated, each experiment was conducted in triplicate.

## Results

### Effects of cryopreservation in trehalose on germ cells enriched for SSCs

To begin to determine the effect of trehalose on cryopreservation of SSCs, testis cells enriched for SSCs by MACS selection of Thy-1 positive cells were cultured for several weeks prior to cryopreservation. The purpose of this culture period was to amplify stem cell numbers from the isolated germ cell population and to replicate pre-freezing culture protocols that will be required to amplify SSC numbers after acquisition of donor tissue in a clinical setting.

**Figure 1:**
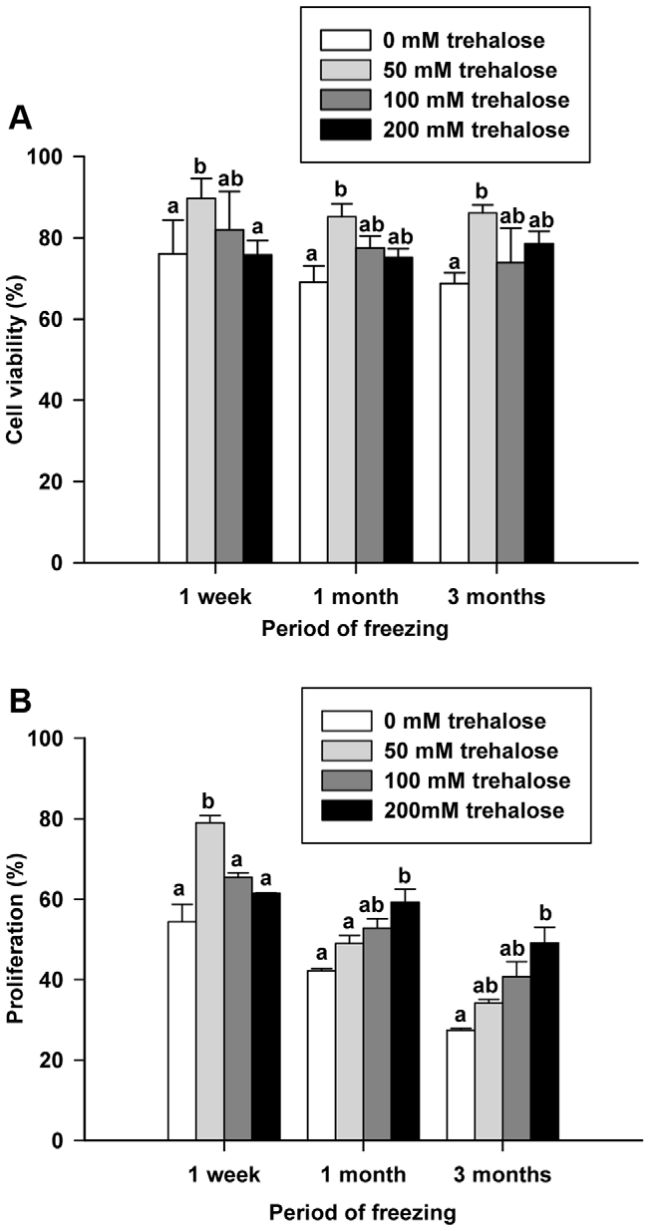
Effects of trehalose on viability and proliferation of SSC enriched testis cells after thawing. (A) Percentage of viable cells after thawing. (B) Percentage of proliferation capacity of frozen germ cells in SSC culture for 1 week after thawing. Figure bars: White: DMSO control group; Light gray: 50 mM trehalose group; Dark gray: 100 mM trehalose group; Black: 200 mM trehalose group. Each treatment group was thawed at 1 week, 1 month, and 3 months post-freezing. Values are means ± SEM (n = 5). Bars within a group with different letters are significantly different (*P*<0.05).

Three different trehalose concentrations (50, 100, and 200 mM in basal freezing media containing DMSO and FBS) were evaluated for their effect on cell viability after thawing of cryopreserved germ cells enriched for SSCs. After 1 week, 1 month, and 3 months frozen cells were thawed and the numbers of viable GFP positive cells were compared to control cells frozen in basal freezing media. Compared to the control freezing group (DMSO only), freezing in 50 mM trehalose resulted in significantly higher percentage of cell viability after thawing at all time-points (76.1±3.4% vs. 89.7±2.0% at 1 week, 69.1±2.3% vs. 85.2±1.8% at 1 month, 68.8±1.5% vs. 86.1±1.2% at 3 months; Figure 1A). In contrast, 100 mM and 200 mM trehalose treatments did not significantly improve cell viability compared to freezing controls.

In addition to viability, thawed cells were also evaluated for their proliferative potential in cell culture 1 week after thawing. Proliferation potential was evaluated as a percentage of proliferation of non-cryopreserved germ cells. All freezing treatments had dramatically lower proliferation rates than non-cryopreserved cells. As seen with cell viability, germ cells enriched for SSCs from the 50 mM trehalose treatment frozen for 1 week had significantly higher proliferation percentage compared to basal freezing media controls (79.0±1.8% vs. 54.4±4.4%); however, no improvement in proliferation was observed for other freezing periods using 50 mM trehalose (Figure 1B). Although freezing in 100 mM trehalose showed no significant improvement in proliferation percentage, 200 mM trehalose significantly improved cell proliferation after freezing for 1 and 3 months compared to basal freezing media controls (59.3±3.2% vs. 42.2±0.6% for 1 month and 49.1±3.9% vs. 27.5±0.5% for 3 months; Figure 1B).

**Figure 2:**
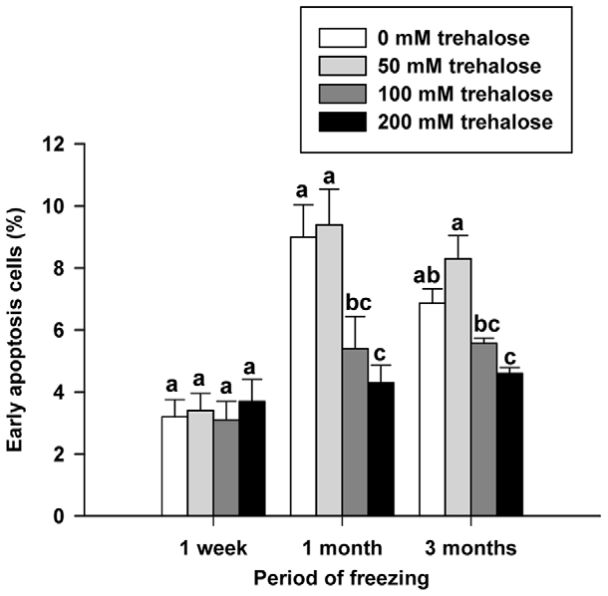
Effects of trehalose on apoptosis of SSC enriched testis cells 12 hours after thawing. Percentage of annexin V binding PI excluding apoptosis positive GFP positive SSC enriched testis cells 12 hours after thawing. Figure bars: White: DMSO control group; Light gray: 50 mM trehalose group; Dark gray: 100 mM trehalose group; Black: 200 mM trehalose group. Each treatment group was thawed at 1 week, 1 month, and 3 months post-freezing. Values are means ± SEM (n = 3). Bars within a group with different letters are significantly different (*P*<0.05).

Because of the contrasting viability and proliferation data, thawed cells were evaluated for apoptosis. Because too low percentage of cells undergoing apoptosis (0.2–0.5%) were observed immediately after thawing regardless of freezing treatment or period (Figure S2), cells were also evaluated at 12 hours after thawing. No difference was observed in the percentage of cells undergoing apoptosis 12 hours after thawing for any treatment after freezing for 1 week. In contrast, after freezing for 1 month, both 100 mM and 200 mM trehalose freezing treatments had significantly lower percentages of apoptotic cells 12 hours after thawing compared to freezing control (9.1±2.1%, 5.4±1.0% and 4.3±1.1% for freezing control, 100 mM trehalose and 200 mM trehalose respectively; Figure 2). Additionally, after freezing for 3 months, 200 mM trehalose treatment had a significantly lower percentage of apoptotic cells 12 hours after thawing compared to control (6.9±0.5% vs. 4.6±0.2%; Figure 2). Collectively, the viability, apoptosis, and proliferation data indicate that 200 mM trehalose treatment is the most effective freezing mechanism for long-term (3 month) preservation of germ cells, including SSCs.

### Long-term assessment of in vitro proliferation

**Figure 3:**
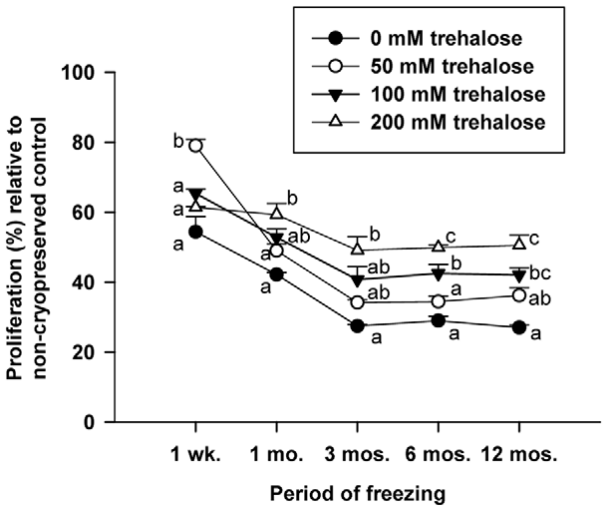
Effects of trehalose on proliferation of SSC enriched germ cells after extended freezing times. Percentage of non-frozen germ cell proliferation capacity of frozen germ cells in SSC culture for 1 week after thawing. No significant differences were observed between proliferation percent after 3, 6, and 12 months of freezing within each treatment group. Figure symbols: Black circle: DMSO control group; White circle: 50 mM trehalose group; Black triangle: 100 mM trehalose group; White triangle: 200 mM trehalose group. Each treatment group was thawed at 1 week, 1 month, 3 months 6 months, and 12 months post-freezing. Values are means ± SEM (n = 5). Different letters indicate significant difference (*P*<0.05) among groups within each freezing period.

Initial data indicated that proliferative activity of germ cells enriched for SSCs in vitro post thawing gradually diminished from 1 week to 3 months of freezing. To determine if diminishing proliferations continued past 3 months freezing time, cells were evaluated for proliferation after 6 and 12 months of freezing. No significant differences were observed between 3, 6 and 12 month freezing periods within each treatment group (Figure 3). As observed with 1 month and 3 month freezing periods, germ cells enriched for SSCs frozen in 200 mM trehalose had significantly higher percentages of proliferative cells compared to controls after both 6 and 12 months of freezing (49.9±0.8% vs. 28.0±1.3% for 6 month and 50.0±1.0% vs. 27±1.0% for 12 months; Figure 3).

### Effects of 200 mM trehalose on SSC activity

**Figure 4:**
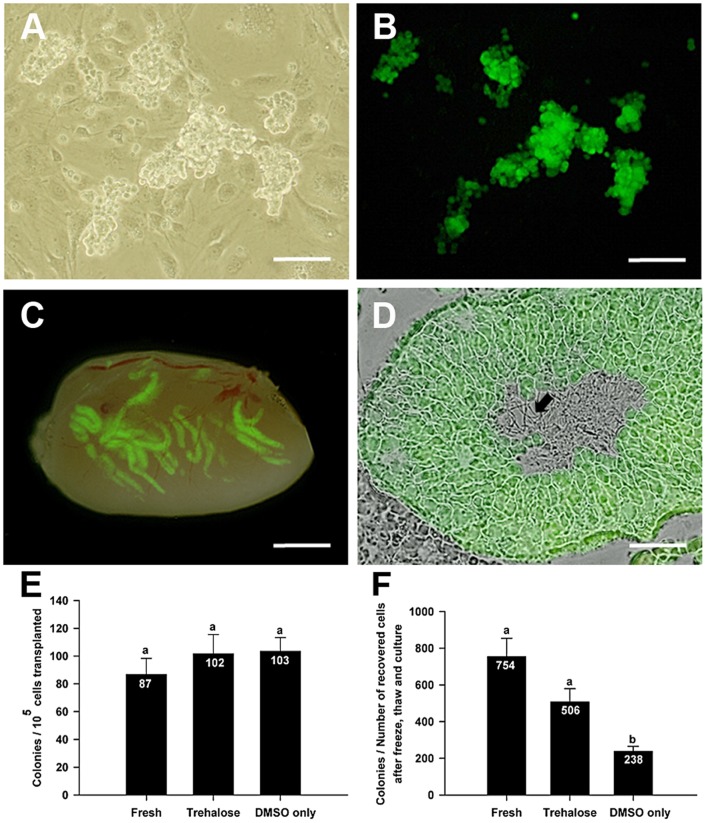
Effects of trehalose on stem cell activity after thawing. (A–B) Bright-field (A) and dark-field fluorescence (B) images of germ cells from GFP positive donors frozen for 3 months in 200 mM trehalose after thawing and 7 days in vitro. (C) Dark-field fluorescence images are over-layed on the bright field image of the same section of a recipient testis transplanted with germ cells from GFP positive donors frozen for 3 months in 200 mM trehalose after thawing and 7 days in vitro culture. Colonies of donor spermatogenesis are distinct green regions of the recipient seminiferous tubules. The number of colonies is directly proportional to the number of SSCs in the transplanted cell population. (D) Cryosections of donor derived germ cell colonies. Green image showing multiple layers of green donor germ cells is over-layed on the bright field image of the same section. Presence of sperm (arrow) in the lumen of the seminiferous tubules indicates complete spermatogenesis. (E) The number of colonies per 10^5^ transplanted cells. (F) The number of colonies per total number of cells recovered after freeze, thaw, and culture. Fresh: non-cryopreserved cells (n = 3 samples; 9 total mice and 16 total testes were transplanted); Trehalose: donor cells frozen with 200 mM trehalose (n = 3 samples; 9 total mice and 16 total testes were transplanted); DMSO: donor cells frozen in control basal freezing media (DMSO only; n = 3 samples; 8 total mice and 14 total testes were transplanted). Values are means ± SEM. Points with different superscripts are significantly different (*P*<0.05). Scale Bars: (A, B)  = 100 µm; (C)  = 2 mm; (D)  = 200 µm.

Evaluation of viability, apoptosis, and in vitro proliferation did not directly evaluate the effects of freezing on the ability of the SSC to generate donor colonies after transplantation to recipient testes. To evaluate the direct effect of cryopreservation with trehalose on the SSC, germ cells that had been frozen for 3 months in control basal freezing media and 200 mM trehalose freezing media were cultured for 7 days (Figure 4A–B) after thawing and subsequently transplanted into recipient testes using the germ cell transplantation technique. Non-cryopreserved, cultured SSCs of the same passage number as the cryopreserved SSCs were also transplanted. This transplantation technique is the only definitive way to directly quantify SSC activity. Two months after transplantation, stem cell content was quantified by counting donor derived colonies of spermatogenesis (Figure 4C). Furthermore, complete spermatogenesis was apparent in donor colonies of spermatogenesis from SSCs frozen in 200 mM trehalose (Figure 4D). Number of colonies per 10^5^ SSCs transplanted was not significantly different between non-cryopreserved SSCs (86.8±11.6) and both freezing treatments (101.7±13.9 and 103.4±10.0 for 200 mM trehalose and DMSO only respectively; Figure 4E) indicating that the ratio of SCC to non-SCC in germ cell cultures was not different between cryopreserved and non-cryopreserved cell populations. When normalized to the total number of recovered cells after freeze, thaw and culture, no significant difference was observed in colony number between non-cryopreserved SSCs (754.1±100.4) and SSCs frozen in 200 mM trehalose (506.3±72.8). Additionally, both the non-cryopreserved SSCs and 200 mM trehalose treatment had significantly more colonies per total number of cells recovered after freeze, thaw and culture than cells frozen in DMSO only (237.5±27.0) (Figure 4F).

### Offspring generation from SSCs frozen in 200 mM trehalose

**Figure 5:**
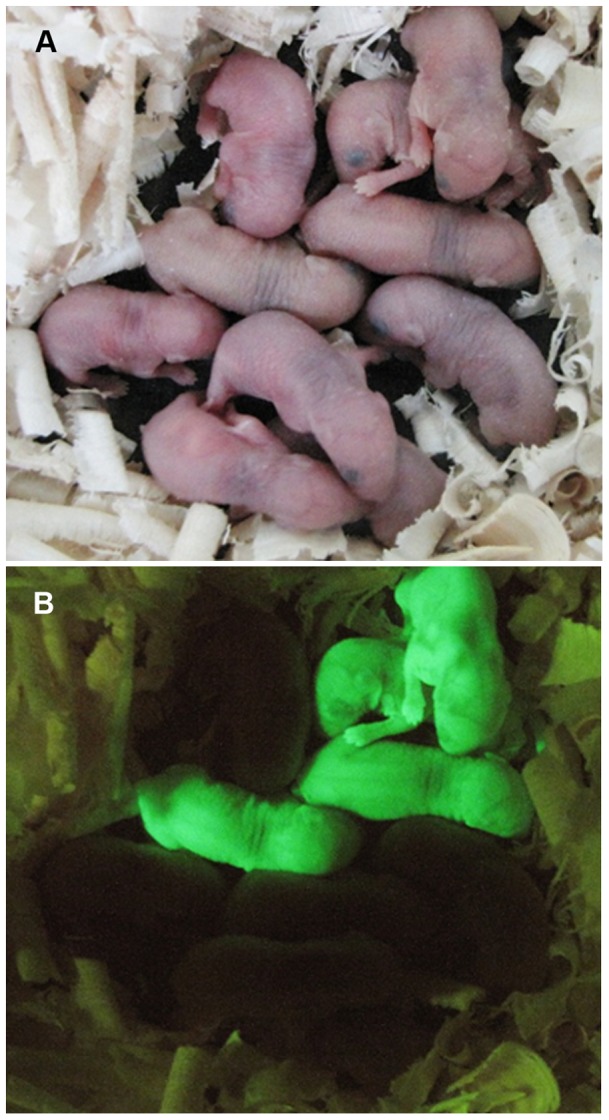
SSCs frozen with 200 mM trehalose have the capacity to generate offspring. (A) Offspring from a C57BL/6 female crossed with a W recipient male (surgery number 685) that was transplanted with SSCs frozen with 200 mM trehalose. (B) The same offspring under UV exposure. Transplanted germ cells are heterozygous for GFP which results in the generation of non-GFP pups from donor cells in addition to GFP+ pups.

To unequivocally prove that SSC function was preserved in the 200 mM trehalose freezing group frozen for more than 3 months, thawed germ cells from C57GFP mice were transplanted into infertile W mice after 1 week of in vitro culture. Several months after transplantation, recipient males were mated with females. Donor derived offspring were produced as evidenced by the presence of green fluorescent expressing GFP-positive pups (Table I; Figure 5A, B).

**Table 1:** Offspring from W recipient mice injected with SSCs frozen in 200 mM trehalose.

Recipient^a^	Recipient age (days)	Freezing period (months)	No. of donor cells injected/testis	Days to first progeny^b^
671 R	12	3	100000	138
681 R, L	24	6	200000	175
685 R	14	6	125000	91

a R, Right testis; L, Left testis. Cells were transplanted into the right testis of immature W recipients, and both testes of mature recipients.

b Days from transplantation to first birth of offspring.

## Discussion

Utilization of SSC culture and transplantation to treat human infertility will require efficient methods of long-term SSC preservation. The present study revealed that cryopreservation is effective for long-term preservation of SSCs and addition of trehalose to basal freezing medium containing DMSO improves stem cell survival after thawing. Trehalose is a nonreducing disaccharide of glucose produced by anhydrobiotic organisms to prevent cell desiccation [42]. Inclusion of trehalose in cryopreservation media has been shown to enhance cell viability of fetal skin cells and fibroblasts [34]–[35] and to increase colony formation of human hematopoietic stem cells [36] after thawing. Trehalose also improves the viability of mammalian germ cells, spermatozoa and oocytes after cryopreservation [37]–[38]. Presumably, the mechanism of action of trehalose is to stabilize cell membranes and associated membrane proteins during the cryopreservation process [34].

In the present study we initially evaluated the effects of control basal freezing medium (containing DMSO and FBS) and basal freezing medium containing 50 mM, 100 mM, and 200 mM trehalose on post-thaw viability of germ cells enriched for SSCs after 1 week, 1 month, and 3 months freezing time. Compared to freezing in DMSO only, the only treatment that resulted in significantly higher post-thaw cell viability was the 50 mM trehalose group (Figure 1A). Higher concentrations of trehalose had numerically, but not significantly higher percentages of viable cells compared to control. These initial data indicated that 50 mM trehalose was the most appropriate treatment examined for freezing of germ cells; however, viability does not directly evaluate cell function.

To confirm functionally which treatment was the best cryoprotectant, thawed germ cells which were enriched for SSCs were maintained in SSC culture for 1 week and assayed for cell proliferation compared to basal medium controls. Proliferation capacity of cells, SSCs and early spermatogonia, is essential for long-term culture maintenance and indicates the presence of SSCs [14]–[15], and was evaluated as a percentage of proliferation of non-cryopreserved germ cell cultures. As expected, regardless of cryopreservation method, freezing resulted in reduced proliferation capacity compared to non-cryopreserved controls. When comparing the different cryopreservation treatments, cells frozen in 50 mM trehalose only had significantly improved proliferation rates after freezing for 1 week compared to the basal freezing media control, however, cells frozen in 200 mM trehalose for 1 month and 3 months had significantly improved proliferation rates compared to basal freezing media control (Figure 1B). These data indicate that although cell viability may not be improved, high concentrations of trehalose improve functionality of mitotically capable cells including SSCs in the frozen sample compared to DMSO alone.

It has been reported that prevention of apoptosis is a potential mechanism by which cryoprotectants protect cells [43]. Thus, we hypothesized that although 50 mM trehalose increased viability directly after thawing, many of these cells had initiated apoptosis, causing the lack of improvement in proliferation after 1 week in culture. To evaluate this hypothesis, we carried out apoptosis assays of thawed cells immediately after thawing and after 12 hours of culture. Because, Annexin V has a high affinity for phosphatidylserine which is translocated from the inner leaflet of the plasma membrane to the outer leaflet in the early phases of apoptosis [44]–[48], we chose the 12 hour period to ensure that thawed cells had enough time to progress into a detectable apoptotic state. As expected, very low percentage of apoptotic cells (0.2–0.5%) was observed among the concentrations of trehalose immediately after thawing regardless of freezing period. When observing cells 12 hours after thawing, no difference was observed in the percentage of apoptotic cells between any treatments frozen for 1 week. Interestingly, an inverse relationship between trehalose concentration and percentage of apoptotic cells after 12 hours in culture was observed for cells frozen for 1 month and 3 months (Figure 2). In fact, cells frozen in 200 mM trehalose had a significantly lower percentage of apoptotic cells 12 hours after thawing than both basal freezing media control and 50 mM trehalose groups.

Collectively, viability, proliferation and apoptosis data indicate that although viability of cells frozen for 1–3 months in 50 mM trehalose immediately after thawing is higher than cells frozen in 200 mM trehlaose, a higher percentage of cells frozen in 50 mM had initiated apoptosis by 12 hours after thawing. This increase in apoptotic cells resulted in the significantly lower proliferation capacity of cells frozen in 50 mM trehalose compared to cells frozen in 200 mM trehalose. Lack of this pattern after 1 week of freezing is most likely due to very low levels of apoptosis being initiated after short term cryopreservation coupled with a lower initial toxicity of 50 mM trehalose compared to 200 mM trehalose. The initial population of germ cells from cell cultures consisted of both SSCs that exhibit a coordinated balance between self-renewal and differentiation [49]–[50] and spermatogonia committed to differentiate. Thus, these data could also indicate that differentiated spermatogonia and SSCs have different tolerances to both freezing and trehalose within certain limits; 400 mM trehalose caused low proliferation and viability (data not shown).

In this study, cell viability remained relatively constant at all freezing periods; however, cell proliferation capability steadily decreased from 1 week to 3 months frozen in liquid nitrogen (Figure 1A–B). Additionally, viability levels were consistent with those previously reported for cryopreservation of mouse SSCs for 5 months [18], but significantly lower than reported for crypreservation of mouse SSCs for 14 years [19]. However, the latter group [19] attributed the low viability to lack of development of efficient cryopreservation techniques at the onset of their freezing period. Interestingly, theoretical models have speculated that cryostability exists at −196°C provided that photophysical events such as free radical formation due to ionizing radiation and cosmic rays are minimized. Given that background radiation is typically 0.1 rad/y frozen cells should be stable for thousands of years [51]. To confirm that our proliferation capability become stable during the freezing process, we evaluated the proliferation activity of cells frozen for 6 and 12 months. As expected, proliferation rate became stable after a 3 month freezing period (Figure 3). These data demonstrate the utility of 200 mM trehalose freezing media to maintain cell activity for periods of time when cells are frozen for greater than 3 months. Additionally, the data also indicates that deleterious effects, such as initiation of apoptosis, are established within the first 3 months of freezing.

In vitro proliferation analysis only indirectly quantifies the effects of cryopreservation on SSC function. The only definitive way to quantify SSC numbers is through the SSC transplantation assay. Non-cryopreserved germ cells and germ cells frozen for 3 months in control basal freezing media and 200 mM trehalose freezing media were transplanted into recipient testes after thawing and 1 week of culture. No difference in colony number per 10^5^ SSCs transplanted between groups was observed (Figure 4E) indicating that SSCs retain their colonization capacity after freezing, and that the ratio of SSC to non-SSC after one week in culture is equivalent between groups. However, when normalized to total number of recovered cells after freeze, thaw and culture, SSCs frozen in DMSO generated significantly fewer colonies than either non-cryopreserved SSCs or SSCs frozen in trehalose, which were not significantly different (Figure 4F). Lack of a difference in germ cell colonization rates normalized to the number of cells recovered between the 200 mM trehalose group and non-cryopreserved cells was unexpected because although the 200 mM trehalose group had a higher percentage of proliferating cells than the other concentration groups, they only have approximately 50% of the proliferative capacity of freshly isolated non-frozen cells. Thus, we hypothesized that these data indicate that the ratio of SSC to non-SSC is higher in cultures established from cryopreserved cells, at the time of culture establishment. Based on viability, proliferation, apoptosis and transplant data, it appears that the SSC is more resistant to the negative effects of cryopreservation than other germ cells, such as differentiating spermatogonia, in the frozen cell population. This is consistent with the overall resistance of the SSC to other agents that damage the testis; the SSC is the last cell type to be destroyed following irradiation or chemical insult [13], [52].

The most definitive test of SSC function is to determine if they have the capacity to give rise to sperm capable of fertilizing an egg to produce offspring. Cells frozen in 200 mM trehalose freezing media for more than 3 months were injected into the testes of recipient W mice. Regardless of recipient age, all W recipients were able to generate offspring through natural mating, although older recipients took longer to produce pups (Table I, Figure 5C–5D). This agrees with previous work demonstrating the immature W testes have 10-fold higher colonization capacity and facilitate faster growth of donor colonies of spermatogenesis compared to mature W testes [13], [53]. Collectively, production of offspring demonstrates that cryopreservation of SSCs using freezing media containing 200 mM trehalose is an effective method for long-term SSC preservation.

The present study was designed to evaluate the effect of the sugar trehalose on cryoporeservation of SSCs. It is possible that other sugars may provide similar protection. Other disaccharides increase cell survival when used with DMSO as a cryoprotectant or lyoprotectant for a variety of cell types [54] including germ cells. Sucrose enhanced the post-thaw survival of bovine type A spermatogonia however, because calves were not produced from the frozen SSCs, definitive evaluation of any potential negative effects of sucrose on the undifferentiated spermatogonia was not conducted [30]. In contrast, inclusion of sucrose to DMSO based freezing media did not provide any increase in post-thaw viability of rodent germ cells [19]. Thus, additional studies are needed to further explore other potential SSC cryoprotectants.

Cryopreservation of SSCs is significantly improved when frozen in freezing media containing DMSO, FBS, and 200 mM trehalose. Thawed SSCs had the capacity to proliferate in vitro, and form colonies of spermatogenesis after transplantation to recipient testes. Additionally, transplanted SSCs were capable of restoring fertility in infertile recipients and give rise to normal offspring. In conclusion, cryopreservation of SSCs in DMSO, FBS, and 200 mM trehalose is a convenient effective mechanism for long-term preservation of the male germ line.

## Supporting Information

Figure S1 **Recovery rate of viable cells after freeze, thaw and wash.** After freezing for 1 week, 1 month, or 3 months, frozen cells (regardless of freezing protocol) were thawed by incubation in a 37°C water bath for 2.5 minutes. After thawing, cells were diluted 1∶10 with MEM alpha containing 10% FBS in a dropwise manner and were centrifuged at 600 g for 7 min at 4°C. The pellet was resuspended in mSFM. Cell viability was determined by trypan blue exclusion. The recovery rate (%) of viable cells was calculated as follows: number of recovered viable cells after freeze, thaw and wash X 100/number of frozen cells (5×10^5^ cells). Compared to the control group (0 mM trehalose), freezing in 50 mM trehalose resulted in significantly higher recovery rate at all time-points (58.2±3.6% vs. 82.9±0.9% at 1 week, 53.9±4.4% vs. 85.1±1.3% at 1 month, 58.3±0.9% vs. 70.7±2.9% at 3 months). In contrast, 100 mM and 200 mM trehalose treatments did not significantly improve recovery rate compared to controls except 100 mM trehalose treatment at 1 month (70.2±2.8% vs. 54.0±4.4%). Figure bars: White: DMSO control group; Light gray: 50 mM trehalose group; Dark gray: 100 mM trehalose group; Black: 200 mM trehalose group. Each treatment group was thawed at 1 week, 1 month, and 3 months post-freezing. Values are means ± SEM (n = 5). Bars within a group with different letters are significantly different (*P*<0.05).
(DOCX)

Figure S2 **Effects of trehalose on apoptosis of SSC enriched testis cells immediately after thawing.** Percentage of annexin V binding PI excluding apoptosis positive EGFP positive SSC enriched testis cells immediately after thawing. Figure bars: White: DMSO control group; Light gray: 50 mM trehalose group; Dark gray: 100 mM trehalose group; Black: 200 mM trehalose group. Each treatment group was thawed at 1 week, 1 month, and 3 months post-freezing. Values are means ± SEM (n = 3).
(DOCX)
