# Supplementary material for: Cryopreservation in Trehalose Preserves Functional Capacity of Murine Spermatogonial Stem Cells
Source: PLoS One. 2013 Jan 22;8(1):e54889. doi: 10.1371/journal.pone.0054889 (PMC3551902; doi:10.1371/journal.pone.0054889)
Supplement: Figure S1 — Recovery rate of viable cells after freeze, thaw and wash. After freezing for 1 week, 1 month, or 3 months, frozen cells (regardless of freezing protocol) were thawed by incubation in a 37°C water bath for 2.5 minutes. After thawing, cells were diluted 1∶10 with MEM alpha containing 10% FBS in a dropwise manner and were centrifuged at 600 g for 7 min at 4°C. The pellet was resuspended in mSFM. Cell viability was determined by trypan blue exclusion. The recovery rate (%) of viable cells was calculated as follows: number of recovered viable cells after freeze, thaw and wash X 100/number of frozen cells (5×10^5^ cells). Compared to the control group (0 mM trehalose), freezing in 50 mM trehalose resulted in significantly higher recovery rate at all time-points (58.2±3.6% vs. 82.9±0.9% at 1 week, 53.9±4.4% vs. 85.1±1.3% at 1 month, 58.3±0.9% vs. 70.7±2.9% at 3 months). In contrast, 100 mM and 200 mM trehalose treatments did not significantly improve recovery rate compared to controls except 100 mM trehalose treatment at 1 month (70.2±2.8% vs. 54.0±4.4%). Figure bars: White: DMSO control group; Light gray: 50 mM trehalose group; Dark gray: 100 mM trehalose group; Black: 200 mM trehalose group. Each treatment group was thawed at 1 week, 1 month, and 3 months post-freezing. Values are means ± SEM (n = 5). Bars within a group with different letters are significantly different (P<0.05). (DOCX) [file pone.0054889.s001.docx]

**Supporting information, Figure S1**


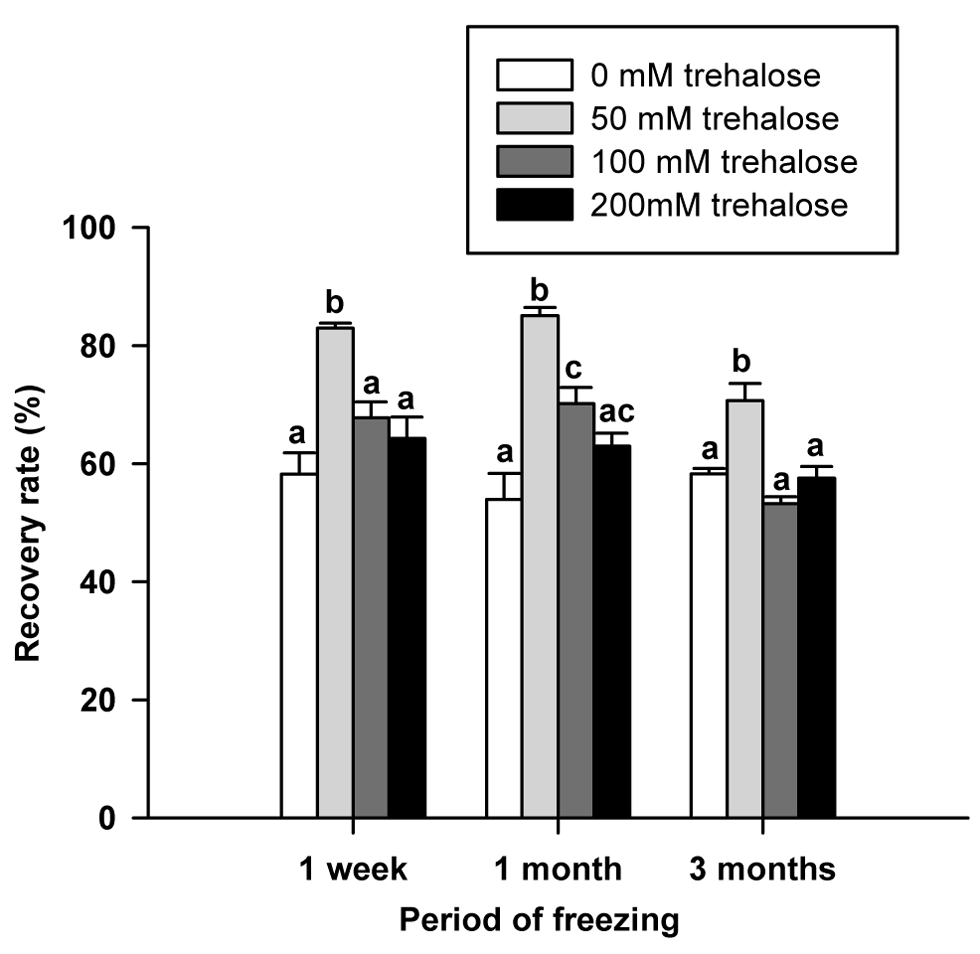


**Figure S1. Recovery rate of viable cells after freeze, thaw and wash.** After freezing for 1 week, 1 month, or 3 months, frozen cells (regardless of freezing protocol) were thawed by incubation in a 37°C water bath for 2.5 minutes. After thawing, cells were diluted 1:10 with MEM alpha containing 10% FBS in a dropwise manner and were centrifuged at 600g for 7 min at 4°C. The pellet was resuspended in mSFM. Cell viability was determined by trypan blue exclusion. The recovery rate (%) of viable cells was calculated as follows: number of recovered viable cells after freeze, thaw and wash X 100 / number of frozen cells (5 X 10^5^ cells). Compared to the control group (0 mM trehalose), freezing in 50mM trehalose resulted in significantly higher recovery rate at all time-points (58.2 ± 3.6% vs. 82.9 ± 0.9% at 1 week, 53.9 ± 4.4% vs. 85.1 ± 1.3% at 1 month, 58.3 ± 0.9% vs. 70.7 ± 2.9% at 3 months). In contrast, 100mM and 200mM trehalose treatments did not significantly improve recovery rate compared to controls except 100 mM trehalose treatment at 1 month (70.2 ± 2.8% vs. 54.0 ± 4.4%). Figure bars: White: DMSO control group; Light gray: 50 mM trehalose group; Dark gray: 100mM trehalose group; Black: 200mM trehalose group. Each treatment group was thawed at 1 week, 1 month, and 3 months post-freezing. Values are means ± SEM (n = 5). Bars within a group with different letters are significantly different (*P* < 0.05).
