# Supplementary material for: Cryopreservation in Trehalose Preserves Functional Capacity of Murine Spermatogonial Stem Cells
Source: PLoS One. 2013 Jan 22;8(1):e54889. doi: 10.1371/journal.pone.0054889 (PMC3551902; doi:10.1371/journal.pone.0054889)
Supplement: Figure S2 — Effects of trehalose on apoptosis of SSC enriched testis cells immediately after thawing. Percentage of annexin V binding PI excluding apoptosis positive EGFP positive SSC enriched testis cells immediately after thawing. Figure bars: White: DMSO control group; Light gray: 50 mM trehalose group; Dark gray: 100 mM trehalose group; Black: 200 mM trehalose group. Each treatment group was thawed at 1 week, 1 month, and 3 months post-freezing. Values are means ± SEM (n = 3). (DOCX) [file pone.0054889.s002.docx]

**Supporting information, Figure S2**


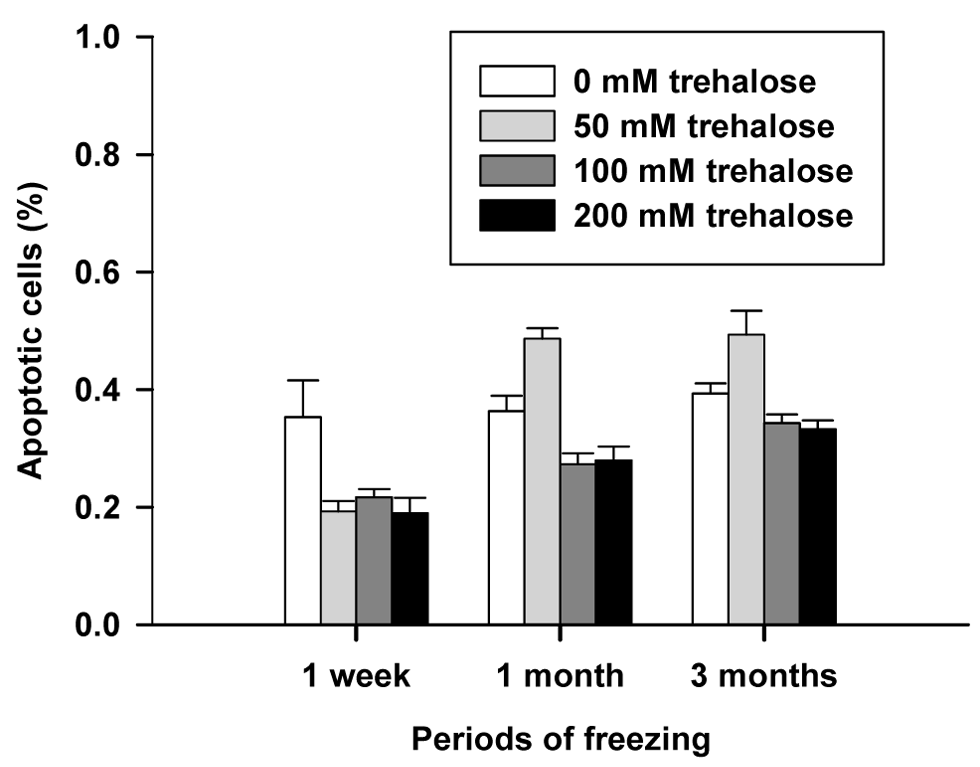


**Figure S2. Effects of trehalose on apoptosis of SSC enriched testis cells immediately after thawing.** Percentage of annexin V binding PI excluding apoptosis positive EGFP positive SSC enriched testis cells immediately after thawing. Figure bars: White: DMSO control group; Light gray: 50 mM trehalose group; Dark gray: 100mM trehalose group; Black: 200mM trehalose group. Each treatment group was thawed at 1 week, 1 month, and 3 months post-freezing. Values are means ± SEM (n = 3).
